# Development of a UPLC-MS/MS Method for Tracking Polymyxin B Dynamics in Soil Inoculated with *Paenibacillus polymyxa*

**DOI:** 10.3390/biom15121694

**Published:** 2025-12-04

**Authors:** Siyu Huang, Xiaorui Li, Xin Lu, Biao Kan

**Affiliations:** National Key Laboratory of Intelligent Tracking and Forecasting for Infectious Diseases, National Institute for Communicable Disease Control and Prevention, Chinese Center for Disease Control and Prevention, Beijing 102206, China; hsy421an@163.com (S.H.); lixiaorui@icdc.cn (X.L.)

**Keywords:** *Paenibacillus polymyxa*, polymyxin B, UPLC-MS/MS, ecological risk

## Abstract

Polymyxins, including polymyxin B (PMB), are last-resort antibiotics against multidrug-resistant Gram-negative infections in humans and livestock. Residual polymyxins from wastewater and manure can accumulate in soil, facilitating the emergence and spread of polymyxin resistance. *Paenibacillus polymyxa*, a natural polymyxin producer used in crop cultivation, may increase soil polymyxin burden. Since PMB strongly adsorbs to soil, its reliable quantification has been challenging. To address this, the extraction solvent and solid-phase extraction procedure were optimized to improve recovery and reduce matrix effects. We developed and validated a UPLC-MS/MS method to quantify PMB in soil. The method showed linearity (10–1000 ng/g), with a limit of detection of 0.86 ng/g and a limit of quantification of 2.12 ng/g. Method validation confirmed acceptable analytical performance. A 28-day monitoring of PMB in soil inoculated with varying *P. polymyxa* doses revealed a dose-dependent increase over the first 14 days, followed by a decline; PMB remained detectable on day 28. Ecological risk assessment using the risk quotient (RQ) indicated that PMB levels in the high-dose group (2 × 10^8^ CFU/100 g) approached the high-risk threshold (RQ ≥ 1) on day 14, while lower doses posed low to medium risk. This work provides a soil PMB quantification method and insight into the ecological risk of *P. polymyxa* application.

## 1. Introduction

The overuse and misuse of antimicrobial drugs is increasingly leading to a rise in the global threat of antimicrobial resistance (AMR) [1]. By 2050, antibiotic resistance is estimated to cause up to 10 million deaths annually, which will far exceed the current estimate of about 700,000 deaths per year [2]. The widespread emergence of multidrug-resistant (MDR) and extensively drug-resistant (XDR) bacteria has made the selection and use of effective antibiotics increasingly challenging [3]. Polymyxins [polymyxin B (PMB) and polymyxin E (PME or colistin)] are associated with dose-dependent nephrotoxicity and neurotoxicity in humans and animals, which mainly manifest as acute kidney injury and neuromuscular disturbances [4,5]. Nevertheless, they have been reintroduced as the last line of defense against MDR Gram-negative pathogens [6]. A substantial proportion of globally produced polymyxins is currently used in food-producing animals (i.e., cattle and swine) for prophylactic, therapeutic, and growth promotion purposes [7]. Under the One Health framework, the surge in polymyxin resistance driven by its intensive usage poses a serious threat to the human–animal–environment ecosystem [8]. Initially, polymyxin resistance was believed to be solely chromosome-mediated and thus less likely to be transmitted [9]. However, the discovery of the first plasmid-mediated mobile colistin resistance (*mcr-1*) gene revealed an exponential increase in the global prevalence and dissemination of polymyxin resistance [10]. To date, *mcr-1* and its nine homologs (*mcr-2* to *mcr-10*) have been found disseminated worldwide and detected across humans, animals, and environmental reservoirs in over 60 countries [11]. The extensive utilization of polymyxins in medical and agricultural sectors acts as a potential source of AMR risk in soil environments. Evidence indicates that polymyxins are incompletely absorbed in humans and livestock, including sheep, cattle, and poultry [6,12]. A significant fraction of polymyxins is released into environmental matrices (clinical wastewater and animal manure) via the fecal and urinary routes. In many countries, particularly in livestock-producing regions of Asia and Latin America, these polymyxin-contaminated environmental matrices are applied to agricultural soil without proper treatment as part of crop management measures (irrigation or manure). Polymyxin residues in soils can enter both surface and ground waters. They may also be taken up by crops, ultimately returning to humans and livestock via drinking water and the food chain. However, published quantitative data on polymyxin concentrations in agricultural soils, manure, wastewater, and drinking water remain scarce, and environmental guideline values specifically for polymyxins in these matrices have not yet been established. As a central reservoir in this cycle, polymyxin-contaminated soils, together with associated AMR risks, have become a growing concern worldwide.

Soil is the largest reservoir of antibiotic-resistant bacteria (ARB) and antibiotic resistance genes (ARGs) [13]. In addition to the direct introduction of ARB and ARGs into soils through agricultural practices such as manure fertilization and wastewater irrigation [14,15], clinically and agriculturally used antibiotics exert selective pressure that further promotes ARB and ARG emergence and spread in soil [16]. Researchers have found a positive correlation between PME usage and the emergence and transfer of polymyxin resistance genes in livestock and soil environments [17,18,19]. A study on swine feedlots showed that continuous PME use resulted in soil residues and elevated *mcr-1* abundance, and these effects were further disseminated to agricultural soils through manure application [20,21]. In addition, several pot experiments have demonstrated that exogenous addition of PMB or PME significantly elevated the total abundance of ARGs in soil and the risk of horizontal gene transfer (HGT) of ARGs [22,23]. These reports suggest that monitoring polymyxin levels in soil will help assess the risk of polymyxin resistance in soil environments at an early stage.

A comprehensive understanding of the source of polymyxins in soil is crucial for reducing their residues and alleviating polymyxin resistance risk. Although the ban on PME use in food-producing animals has indirectly reduced the residues in soil, another potential source of polymyxins has been overlooked for a long time. *Paenibacillus polymyxa* is a Gram-positive, rod-shaped, non-pathogenic, spore-forming bacterium widely distributed in plant roots. *P. polymyxa* exhibits multiple functions, such as nitrogen fixation, phosphate solubilization, and the synthesis of plant growth hormones and antimicrobial compounds [24]. It is combined with carriers such as peat and diatomaceous earth to form microbial inoculant, which promotes crop growth and controls plant diseases and pests [25]. Moreover, *P. polymyxa* has a strong antibiotic biosynthetic capacity, with polymyxins being its most well-known secondary metabolite [24]. Most polymyxins used in clinical practice were initially extracted from *P. polymyxa* fermentation broths. Previous researchers have assessed the effects of *P. polymyxa* on maize growth, fusarium wilt control, and nematode communities [26,27,28]. However, the effects of its secreted polymyxins on soil microorganisms and ARGs remain understudied. Therefore, quantifying soil polymyxin levels after *P. polymyxa* inoculation is essential for evaluating the safety of *P. polymyxa* and resistance risk in agricultural soils.

With the continuous development of diverse analytical techniques, the methods used to detect and quantify polymyxins have evolved from low-sensitivity ones, such as thin-layer chromatography and capillary electrophoresis, to high-sensitivity techniques like high-performance liquid chromatography-ultraviolet (HPLC-UV) and liquid chromatography-mass spectrometry (LC-MS). Currently, liquid chromatography-tandem mass spectrometry (LC-MS/MS) methods are widely used to quantify polymyxins in various matrices, including plasma, meat, milk, and water [29,30,31,32,33,34,35]. These techniques demonstrate high accuracy and sensitivity. However, methods for determining polymyxins in soil matrices, which are highly complex, remain underdeveloped. To date, only one study has reported a method combining solid-phase extraction (SPE) with ultraperformance liquid chromatography-tandem mass spectrometry (UPLC-MS/MS) for determining PME in soils [36]. Although this study provided valuable reference for polymyxins analysis in complex soil matrices, its primary focus was the adsorption, desorption, and degradation behavior of PME. It did not include the systematic development of quantitative methods for PMB in soil. While PMB and PME share a common peptide backbone, they differ in hydrophobicity, side-chain composition, and soil-adsorption behavior [37]. These differences may affect extraction efficiency and chromatographic performance. Moreover, the environmental accumulation and potential resistance risks of PMB remain insufficiently investigated. Thus, it is necessary to establish a dedicated and robust method specifically for PMB quantification in soil. Such a method is essential for accurately evaluating its environmental behavior and ecological risks.

In this study, we aimed to (i) develop and validate a sensitive and robust UPLC-MS/MS method for quantifying PMB in complex soil matrices, (ii) apply this method to elucidate the temporal dynamics of PMB in soils inoculated with different doses of *P. polymyxa*, and (iii) evaluate the ecological risk of PMB residues in soils using the risk quotient (RQ) method. Overall, our work provided a dedicated quantitative method for PMB in soils and a case study that together offer a practical basis for future monitoring and ecological risk assessment of PMB residues in soils.

## 2. Materials and Methods

### 2.1. Chemicals and Reagents

Polymyxin B sulfate (PMB; a mixture of polymyxin B1 and polymyxin B2) was purchased from INALCO SPA (Milano, Italy), while Polymyxin E sulfate (PME; a mixture of polymyxin E1 and polymyxin E2) was purchased from Biotopped Technology (Beijing, China). The stock solutions of PMB and PME (1 mg/mL) were prepared by dissolving the respective compounds in water and stored at −20 °C. The working standard solutions of PMB and PME were prepared by diluting the stock solution in water just before use. Methanol, formic acid, and acetonitrile of chromatographic grade were obtained from Aladdin Biochemical Technology (Shanghai, China). Phosphate-buffered saline (PBS) was purchased from Invitrogen Corporation (Carlsbad, CA, USA). Luria–Bertani (LB) broth was composed of 1% NaCl, 0.5% yeast extract, and 1% tryptone; for solid medium, 1.5% agar was added. The ingredients for the LB broth were purchased from Oxoid Ltd. (Basingstoke, UK). Ultrapure water used in this study was produced by a Milli-Q Plus water purification system (Millipore, Bedford, MA, USA). The cartridges Cleanert PEP (60 mg, 3 mL) from Agela Technologies (Tianjin, China), Oasis MCX (60 mg, 3 mL) and Sep-Pak C18 (200 mg, 3 mL) from Waters (Milford, MA, USA), and HLB (200 mg, 6 mL) from BKMAMLAB Biotechnology (Changde, China) were used to perform SPE. All other reagents of analytical grade were purchased from Sinopharm Chemical Reagent (Shanghai, China).

### 2.2. P. polymyxa Strains Isolation and Soil Sample Collection

To obtain a PMB-producing strain for the soil inoculation experiments, five *P. polymyxa* microbial inoculants were purchased from different pesticide markets in Beijing, China. Each inoculant was dissolved and diluted in PBS to obtain gradient concentrations, which were then spread onto LB agar plates. Single colonies appearing on these plates were picked and identified as *P. polymyxa* using matrix-assisted laser desorption ionization-time of flight mass spectrometry (MALDI-TOF MS) EXS 2600 from Zybio Inc. (Chongqing, China) and 16S rRNA sequencing performed by Majorbio Co., Ltd. (Shanghai, China). From these inoculants, five *P. polymyxa* isolates were obtained, and an additional reference strain, *P. polymyxa* ATCC 842, was purchased from BIOBW Biotechnology Co., Ltd. (Beijing, China). All six strains were cultured in LB broth for 24 h at 30 °C with shaking. Their LB cultures were subsequently analyzed using UPLC-MS/MS to determine polymyxin subtypes and quantify production levels. The strain that exclusively produced PMB and showed the highest PMB production level was selected for subsequent soil inoculation experiments.

Soil sample were collected using a five-point sampling method from a farmland in Changping District, Beijing, China (40°11′ N, 116°18′ E). The topsoil (0–20 cm) was obtained after removing visible impurities such as stones and grassroots. The soil was then air-dried, passed through a 4.0 mm sieve, and stored at 4 °C until further use. The collected soil was classified as loam, with a neutral pH of 7.41 and a total organic matter content of 23.2 g/kg.

### 2.3. Preparation of PMB-Spiked Soil Samples

For method development and validation, the collected soil samples were divided evenly into 500 g portions and placed in polyethylene plastic containers (14.4 cm × 22.2 cm × 12.5 cm) sterilized previously with 75% ethanol. The PMB stock solution was diluted with Milli-Q water to the desired concentrations and uniformly sprayed onto the soil in the container using an atomizing device, followed by thorough mixing to ensure homogeneous PMB distribution. The final concentration of the exogenously added PMB in the soil was adjusted to 10, 50, and 200 ng/g. Meanwhile, the control group was sprayed with an equal volume of Milli-Q water, and three biological replicates were established for each group. The treated samples in the containers were incubated in a controlled climate chamber (25 °C, 70% relative humidity) under dark conditions. During incubation, the soil moisture content was maintained at approximately 30% (equivalent to 70% of field capacity) by regularly weighing the soil and replenishing it with Milli-Q water [38]. The soil samples were collected 12 h after PMB was spiked into the soil and stored at −20 °C until further analysis.

### 2.4. Extraction of Polymyxin from Soil

Two grams of frozen soil was extracted with 10 mL of 10% trichloroacetic acid: acetonitrile (40:60, *v*/*v*) containing 0.01 M EDTA by incubating at 25 °C and 200 rpm for 2 h. This EDTA level is sufficient to chelate most metal ions, thereby reducing PMB-metal complexation [39]. The mixture was then centrifuged at 12,000 rpm for 10 min, and the supernatant was collected and subjected to liquid–liquid extraction with an equal volume of n-hexane. The resulting aqueous phase was used for subsequent SPE processing.

Waters Oasis MCX SPE cartridge (60 mg, 3 mL) was first preconditioned with 3 mL of methanol and 3 mL of 0.1% formic acid in water. After loading 5 mL of the extract (flow rate ≤ 1 mL/min), the cartridge was washed with 3 mL of 5% methanol in water containing 0.1% formic acid. The target compounds were then eluted with 5 mL of methanol: acetonitrile (2:1, *v*/*v*) containing 2% formic acid (flow rate ≤ 0.5 mL/min). The eluate was evaporated to dryness under vacuum at 50 °C. The residue was redissolved in 0.1% formic acid in water, filtered through a 0.22 μm membrane and subjected to UPLC-MS/MS analysis.

### 2.5. UPLC-MS/MS Analysis

The polymyxins (PMB or PME) in LB broth and soil samples were quantified using an LCMS-8060 triple quadrupole mass spectrometer (Shimadzu, Kyoto, Japan) equipped with an ACQUITY UPLC BEH C_18_ column (2.1 mm × 50 mm, 1.7 μm; Waters, Milford, MA, USA). The column temperature was maintained at 40 °C, and the injection volume was set at 10 μL. The mobile phases consisted of solvent A (acetonitrile with 0.1% formic acid and 2 mM ammonium formate) and solvent B (water with 0.1% formic acid and 2 mM ammonium formate). The gradient elution program was set as follows: 0–0.5 min, 10% A; 0.5–3.0 min, 10–95% A; 3.0–4.5 min, 95% A; and 4.5–6.0 min, 95–10% A. The flow rate was set at 0.4 mL/min.

Mass spectrometric detection was performed using an electrospray ionization (ESI) source in positive ion mode. The optimized ion source parameters were set as follows: nebulizing gas flow, 3.0 L/min; heating gas flow, 10.0 L/min; drying gas flow, 10.0 L/min; interface temperature, 300 °C; desolvation line (DL) temperature, 250 °C; heat block temperature, 400 °C; and desolvation temperature (ion source), 526 °C. Quantification was performed in multiple reaction monitoring (MRM) mode. Full-scan analyses showed that the doubly charged ions [M + 2H] ^2+^ were the most abundant precursor ions for the four polymyxin subtypes (PMB1, PMB2, PME1, and PME2) under the present conditions. Product-ion scans identified a dominant fragment at *m*/*z* 101 (z = +1), originating from the Dab residue, which was selected as the quantifier ion for all subtypes, consistent with previous polymyxin studies [32,40]. The collision energy (CE) was set to 30 eV for all transitions, as determined during method optimization. Concentrations of PMB and PME were determined as the sum of the peak areas of their respective components: PMB1 (*m*/*z* 602.25 > 100.80) and PMB2 (*m*/*z* 595.40 > 101.25) for PMB, and PME1 (*m*/*z* 585.85 > 101.20) and PME2 (*m*/*z* 578.95 > 101.00) for PME. Representative MS/MS spectra of the four polymyxin subtypes are shown in Figure S1. Data acquisition and processing were conducted using LabSolutions software (version 5.97, Shimadzu, Kyoto, Japan).

### 2.6. Method Validation

The selectivity, linearity, limit of detection (LOD), limit of quantification (LOQ), accuracy, precision, recovery and matrix effects (ME) were assessed to validate the developed UPLC-MS/MS method. To determine the selectivity, soil samples without exogenous PMB were used as blank controls. Both blank and PMB-spiked soil samples were analyzed under identical chromatographic conditions to ensure that no interfering peaks (within ± 5% of the target peak area) co-eluted with PMB. The linearity was evaluated using seven matrix-matched calibration standards (10, 20, 40, 100, 200, 500, and 1000 ng/g), prepared by spiking known concentrations of PMB into a single batch of blank soil extract. The peak area responses at each concentration were used to construct the calibration curve. The LOD and LOQ were defined based on signal-to-noise (S/N) ratios of 3 and 10, respectively. Accuracy was evaluated through spiking experiments at three concentrations (10, 50, and 200 ng/g), each analyzed in triplicate, and results falling within 85–115% were deemed satisfactory. Precision was expressed as the relative standard deviation (RSD) and assessed by intra-day (three replicates) and inter-day (three consecutive days) analyses, with RSD values below 15% considered within the allowable range. Recoveries were determined at the same spiking levels, and values between 80 and 120% were regarded as meeting method requirements. Matrix effects were assessed by comparing PMB responses in solvent and blank soil extract. ME values within 85–115% were considered acceptable.

### 2.7. Quantification of PMB in Soil Inoculated with P. polymyxa

The *P. polymyxa* strain was cultured in LB broth at 30 °C overnight with shaking at 180 rpm. The culture was then transferred to sterile tubes and centrifuged at 12,000× *g* for 3 min at 4 °C. The supernatant was removed, and the bacterial pellet was washed three times with PBS (pH 7.2) and resuspended in PBS. Then, *P. polymyxa* suspension was adjusted to the required concentrations using PBS, with OD_600_ = 1.5 corresponding to approximately 10^8^ CFU/mL [41].

Currently, the application of *P. polymyxa* microbial inoculants in agricultural fields lacks standardized guidelines, and the recommended dosages vary considerably depending on the manufacturer, formulation type, application method, target crop, and pest or disease control objectives [42]. To simulate the range of doses used in agricultural practice, six different doses of *P. polymyxa* were applied to soil. The six doses were categorized into three groups: low-dose (0 and 10^2^ CFU/100 g dry soil), medium-dose (10^4^ and 10^6^ CFU/100 g dry soil), and high-dose (10^8^ and 2 × 10^8^ CFU/100 g dry soil). The adjusted *P. polymyxa* suspensions were individually sprayed onto the soil of each treatment using atomizing devices, followed by thorough mixing to ensure homogeneous distribution. The 0 CFU/100 g group was sprayed with an equal volume of PBS, and three biological replicates were established for each treatment. The incubation conditions in the controlled climate chamber were consistent with those used in the PMB-spiked soil process. Soil samples were collected on days 0, 7, 14, 21, and 28 after inoculation and stored at −20 °C for PMB quantification.

### 2.8. Ecological Risk Assessment of PMB in Soil

The ecological risk of PMB in soil was evaluated in accordance with the Technical Guidance Document (TGD) on Risk Assessment of the European Commission (European Commission, 2003) [43], following the equilibrium partitioning method as described by Ren et al. [44]. The risk quotient (RQ) was calculated as follows:

(1) $${RQ=\frac{MEC}{PNEC}}_{soil}$$

where MEC (mg/kg dry soil) represents the measured concentration of PMB in soil inoculated with *P. polymyxa*, and PNEC_soil_ represents the predicted no-effect concentration of PMB in soil. According to the TGD, the PNEC_soil_ of PMB was estimated from the aquatic PNEC_water_ via the soil-water partition coefficient (*K_soil-water_*) as follows:

(2) $${PNEC}_{soil}={PNEC}_{water}\times K_{soil\text{-}water}$$

(3) $$K_{soil\text{-}water}=F_{water\text{-}soil}+F_{solid\text{-}soil}\times \left(K_{Psoil}\times \rho_{solid}/1000\right)$$

where *F_water-soil_* and *F_solid-soil_* are the volume fractions of water and solid phases in the soil, taken as 0.2 and 0.6 in this study, respectively, and ρ_solid_ is the soil solid density (2500 kg/m^3^) as defined in TGD. The solid phase-water distribution coefficient *K_Psoil_* of PMB was approximated using the experimental adsorption data for PME reported by Peng et al. [36], due to the structural and physicochemical similarity between PME and PMB.

Specifically, the *K_Psoil_* value of 2500 L/kg for loam soil was derived from PME’s equilibrium adsorption capacity (*Q*_e_ = 4599.8 ng/g) and desorption rate (0.04%) under an initial aqueous concentration of 1000 ng/mL. Based on these values, the final calculated *K_soil-water_* is 3750.2. Combined with the aquatic PNEC_water_ of PMB (0.06 μg/L) proposed by the AMR Alliance Science-Based PNEC Targets [45], the predicted no-effect concentration of PMB in soil (PNEC_soil_) was estimated to be 0.225 mg/kg dry soil. The risk assessment criteria were categorized into the following three levels: (1) RQ < 0.1, low risk; (2) 0.1 ≤ RQ ≤ 1, medium risk; and (3) RQ > 1, high risk. The calculated RQ values were used to qualitatively and quantitatively assess the potential ecological risk of PMB residues in soil.

### 2.9. Statistical Analysis

Statistical analysis was performed using SPSS (version 20.0, IBM, Armonk, NY, USA), and the graphs were created using GraphPad Prism (version 9.0.0, GraphPad Software, San Diego, CA, USA). Data were represented as mean ± standard deviation (SD). Calibration curve of PMB was constructed using the least-squares method, and the determination coefficient (R^2^) was determined to assess linearity. The differences among the means of multiple groups were determined using one-way analysis of variance (ANOVA) and were considered significant at *p* < 0.05.

## 3. Results

### 3.1. Sample Preparation Optimization, and Method Validation

**Figure 1:**
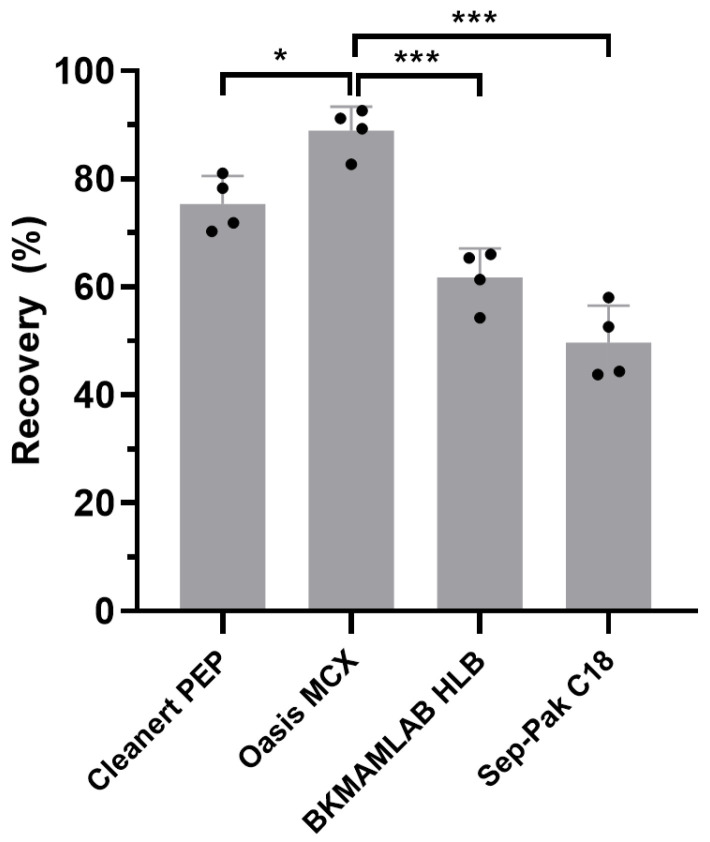
Recovery of PMB from soil using different SPE cartridges. Data are presented as mean ± SD (*n* = 4). One-way ANOVA was used for statistical analysis. Asterisks indicate statistically significant differences between groups (* *p* < 0.05, *** *p* < 0.001).

The present study aimed to develop a UPLC-MS/MS method to quantify the concentration of PMB in soil. Sorbent selection in the SPE process critically affects impurity removal and the extraction efficiency of target compounds. We first tested four different types of commercially available SPE cartridges, namely Cleanert PEP (60 mg), Oasis MCX (60 mg), BKMAMLAB HLB (200 mg), and Sep-Pak C18 (200 mg). All cartridges were activated, rinsed, and eluted according to the manufacturers’ instructions. This approach revealed that Oasis MCX, a mixed-mode cation exchange cartridge, exhibited the highest and most consistent recovery of PMB from soil (Figure 1), and was thus selected for subsequent UPLC-MS/MS method validation.

**Figure 2:**
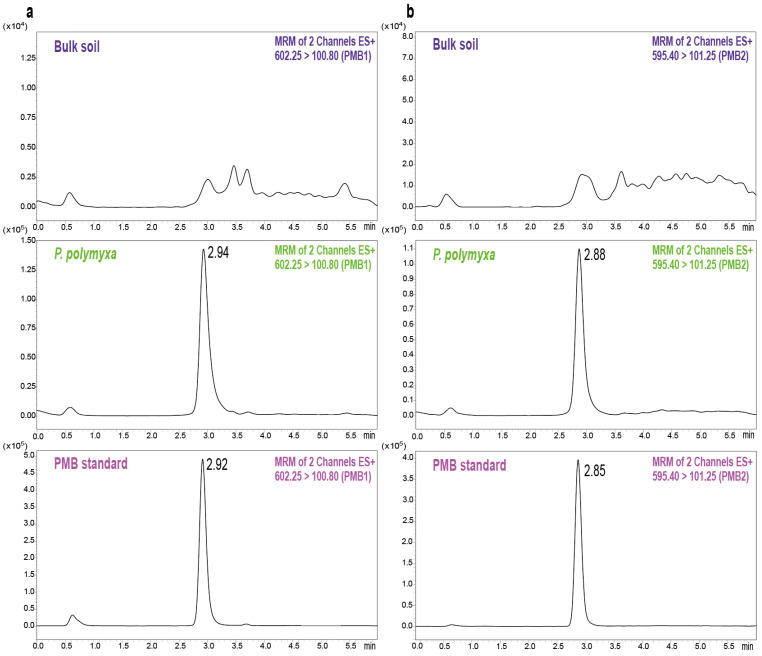
Representative UPLC-MS/MS chromatograms of PMB1 (**a**) and PMB2 (**b**) in blank soil, *P. polymyxa* inoculated soil, and PMB standard solution.

Further, the chromatograms from blank and PMB-spiked soil samples were compared to verify the method’s selectivity. No endogenous interference from soil was observed at the retention time of PMB, confirming its specificity (Figure 2). In the calibration curve of PMB, good linearity was observed for the 10–1000 ng/g concentration range, with determination coefficient (R^2^) exceeding 0.99 (Table 1). The corresponding calibration curve is shown in Figure S2. The LOD and LOQ for PMB were 0.86 ng/g and 2.12 ng/g, respectively, based on spiked blank soil. Subsequently, spiked soil samples at three concentration levels (10, 50, and 200 ng/g) were analyzed to evaluate the accuracy, precision, and recovery of the method. The intra-day accuracy and precision ranged from 96.54% to 104.47% and 1.88% to 3.15%, respectively, while the inter-day values ranged from 97.09% to 100.28% for accuracy and 2.63% to 3.84% for precision (Table 2). The mean recovery in spiked soil samples ranged from 83.58% to 88.83%, which falls within the acceptable criteria. Further comparison of the analyte peak areas of post-extracted blank matrix with those of the pure solvent revealed that the ME values ranged from 87.7% to 90.2% (Table 2), indicating acceptable matrix-related interference for the established method. These observations indicated that the established method is specific, sensitive, and reliable for quantifying PMB in soil matrices, and therefore, suitable for environmental monitoring applications.

### 3.2. Temporal Dynamics of PMB in Soil Inoculated with P. polymyxa

**Figure 3:**
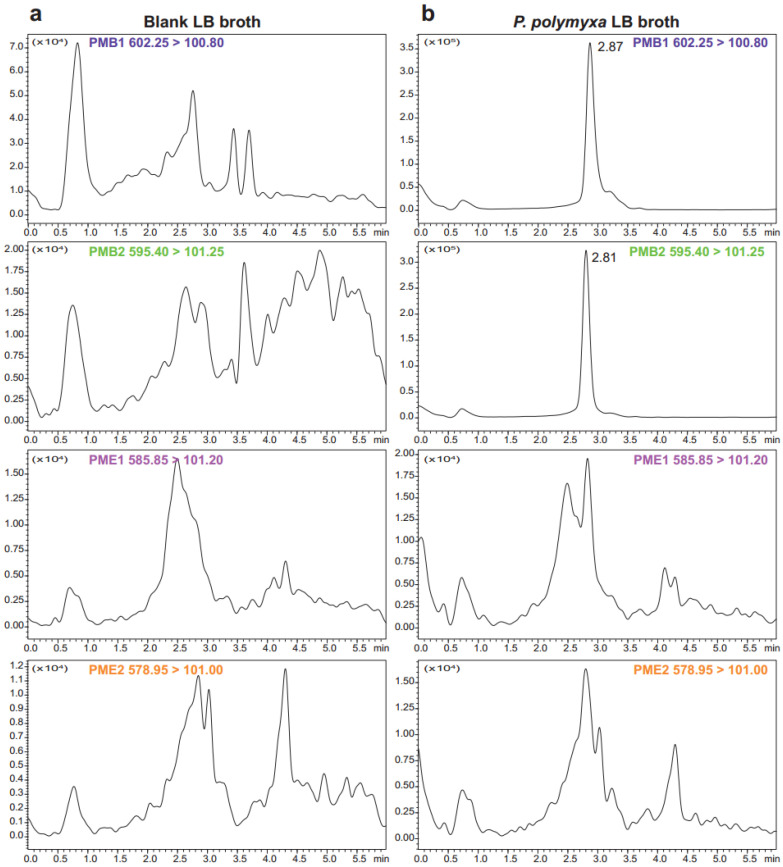
UPLC-MS/MS chromatograms of blank LB broth (**a**) and *P. polymyxa* LB broth (**b**). The method detected PMB components (both PMB1 and PMB2) in the *P. polymyxa* LB broth and revealed no detectable levels of PME (both PME1 and PME2).

To confirm the polymyxin subtype synthesized by the selected *P. polymyxa* strain, UPLC-MS/MS analysis was performed on LB broth after 24 h of incubation at 30 °C with shaking. As shown in Figure 3, this strain exclusively produced PMB, with no detectable PME.

**Figure 4:**
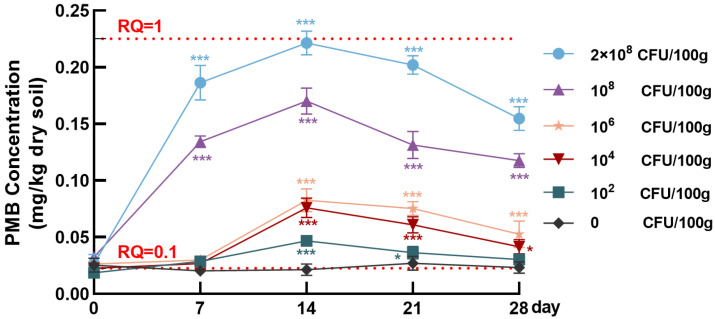
Time-concentration profiles of PMB in soil inoculated with different doses of *P. polymyxa*. Each data point represents the mean ± SD (*n* = 3). One-way ANOVA was used for statistical analysis. Asterisks (*) indicate statistically significant differences between a specific time point and day 0 within a dose group (* *p* < 0.05, *** *p* < 0.001).

Under simulated cultivation conditions, we found a clear dose- and time-dependent dynamics of PMB concentrations in soil after inoculation with six doses of *P. polymyxa* (Figure 4). In the control group (0 CFU/100 g), PMB levels remained consistently low over the 28-day incubation period (0.020–0.027 mg/kg) without significant fluctuation (*p* > 0.05). These levels were far below the natural background levels of PME in soil predicted by Menz et al. [46]. In contrast, the inoculated groups exhibited distinct temporal dynamics in PMB levels. During the initial phase (0–7 days), high-dose groups (10^8^ CFU/100 g and 2 × 10^8^ CFU/100 g) showed a rapid increase in PMB concentrations. Specifically, the 2 × 10^8^ CFU/100 g group exhibited the most pronounced rise, with PMB reaching 0.186 ± 0.015 mg/kg on day 7. The 10^8^ CFU/100 g group showed a lower but still elevated level of approximately 0.134 ± 0.005 mg/kg at the same time. This early increase in PMB levels is likely attributed to the successful colonization of *P. polymyxa* and the initiation of PMB biosynthesis in soil. By contrast, the low- and medium-dose groups maintained a slower accumulation of PMB during this period.

By day 14, two high-dose groups (10^8^ CFU/100 g and 2 × 10^8^ CFU/100 g) reached their respective peak concentrations; the 2 × 10^8^ CFU/100 g group attained the highest level of 0.221 ± 0.011 mg/kg, and the 10^8^ CFU/100 g group reached 0.170 ± 0.012 mg/kg of PMB. In contrast, the medium-dose groups (10^4^ CFU/100 g and 10^6^ CFU/100 g) exhibited a more gradual accumulation of PMB and peak values substantially lower than those observed in the high-dose groups. The low-dose group (10^2^ CFU/100 g) maintained PMB concentrations below 0.05 mg/kg throughout the incubation period (days 0 to 28), showing only minor fluctuations. During the later phase (days 14–28), a drop in PMB concentration was observed across all dose groups. This decrease was most pronounced in the high-dose groups: PMB levels declined by approximately 70.14% in the 2 × 10^8^ CFU/100 g group by day 28, yet remained relatively high at 0.155 ± 0.010 mg/kg; similarly, a 69.41% reduction was observed in the 10^8^ CFU/100 g group. In contrast, the low- and medium-dose groups experienced a more gradual decline, with concentrations stabilizing between 0.023 and 0.053 mg/kg by day 28. These reductions likely reflect decreased metabolic activity and cell viability of *P. polymyxa* over time, leading to a slowdown in PMB biosynthesis and possible degradation or adsorption losses in the soil. Overall, the data suggest that the inoculation dose of *P. polymyxa* and exposure duration jointly determine PMB dynamics in soil. Higher inoculation doses led to more rapid and pronounced PMB accumulation as well as longer environmental persistence. These findings indicate that the application dose of *P. polymyxa* as a microbial inoculant should be carefully determined to mitigate the potential risk of PMB accumulation in soil.

### 3.3. Ecological Risk Assessment of PMB in Soil Inoculated with P. polymyxa

Research on the ecological impact of PMB residues in soil remains limited. Studies have mainly provided qualitative evaluations, focusing on the effects of PMB on soil microbial community and soil macrofauna (e.g., earthworms) [22,47]. To date, no standardized quantitative method has been established to assess the ecological risks posed by PMB in soil environments. In light of this, the current study applied the risk quotient (RQ) method to quantitatively evaluate the ecological risk of PMB in soils inoculated with *P. polymyxa*.

Based on the measured concentrations of PMB and the estimated PNEC_soil_, the RQ values over 28 days were calculated for each *P. polymyxa* dose applied to the soil (Figure 4) in all groups. The temporal trends in RQ values were consistent with those of PMB concentrations across all groups. In the 2 × 10^8^ CFU/100 g group, the RQ value rose sharply during the early phase, surpassing the medium-risk threshold (RQ ≥ 0.1) as early as day 7 (RQ = 0.828) and approaching the high-risk threshold by day 14 (RQ = 0.984). Although PMB levels in this group decreased later, the RQ remained at 0.687 on day 28. The 10^8^ CFU/100 g group exhibited a similar trend, with the RQ value peaking at 0.755 on day 14 and then declining to 0.523 by day 28. In both high-dose groups, RQ values consistently remained within the medium-risk range throughout the 28-day period.

The 10^6^ CFU/100 g and 10^4^ CFU/100 g groups exhibited similar trends, with RQ values peaking at approximately 0.35 on day 14, indicating only minor dose-dependent differences in PMB ecological risk at medium inoculation levels. In contrast, the 10^2^ CFU/100 g group exhibited a brief and slight RQ rise, peaking at 0.208 on day 14 and falling back to 0.135 by day 28, remaining in the medium-risk zone only temporarily. The 0 CFU/100 g group consistently showed RQ values below 0.12, confirming the low ecological risk of PMB in the background soil. These results confirm that inoculation of *P. polymyxa* at high doses can lead to PMB accumulation in soil at levels sufficient to trigger moderate ecological risk for at least three weeks after application. This finding highlights the need for planning *P. polymyxa* dose management strategies and post-application monitoring measures to mitigate unintended PMB burden in agricultural soils.

## 4. Discussion

In the current study, we developed a sensitive and accurate UPLC-MS/MS method to quantify PMB concentrations in soil, helping to address the current lack of reliable analytical approaches for PMB quantification in soil environments. This method revealed a clear accumulation of PMB in soil 28 days after inoculation with *P. polymyxa*. Detailed analysis showed that this accumulation was dependent on both dosage and time after inoculation. In the collected loam soil, higher doses of *P. polymyxa* led to a rapid increase and prolonged persistence of PMB. Finally, the ecological risk assessment of PMB using the RQ method highlighted the potential environmental impact of high-dosage applications of *P. polymyxa*.

Polymyxins are cationic polypeptide antibiotics that tend to adsorb to soil via hydrophilic interactions and hydrogen bonding. Research has proven that PMB has a stronger affinity for soil than PME [37]. Accordingly, an effective method is essential for extracting and enriching PMB from soil for accurate quantification. Considering that PMB and PME share the same peptide backbone, the extraction solvent composition reported by Peng et al. provided a reasonable starting point [36]. However, due to the different metal-binding tendency of PMB, the solvent was further modified with 0.01 M EDTA as a cation chelator to reduce PMB-metal complexation, which led to satisfactory extraction performance [39,48]. Typically, soil matrices are rich in organic acids, micropollutants, and humic substances [49], which are non-selectively co-extracted with PMB during the extraction process. To overcome these challenges related to the matrix, lipid impurities in the extract were first removed by liquid–liquid partitioning with n-hexane. The resulting aqueous extract was purified using mixed-mode strong cation-exchange cartridges (Oasis MCX). In our tests, these cartridges achieved higher PMB recovery than hydrophilic-lipophilic balanced sorbents such as Cleanert PEP and BKMAMLAB HLB [50]. This advantage arises from the strong cation-exchange interaction between protonated PMB and the sulfonic acid groups on MCX under acidic conditions, enabling stronger retention and reducing loss during washing [51]. Although PMB is highly cationic, its amphiphilic structure still allows moderate hydrophobic interaction with C18 phases, explaining the measurable but lowest retention observed among the tested sorbents. In addition, polymyxins exhibit substantial instability in basic and neutral aqueous solutions [52,53]. Therefore, to enhance the stability of PMB during the chromatographic analysis process, 0.2% formic acid was added to both A and B mobile phases. This modification, in combination with gradient elution, significantly minimized peak tailing and improved PMB separation in chromatography. Overall, the linearity, sensitivity, and recovery of the developed PMB method fell within similar low-ng/g ranges to those reported for Peng et al.’s quantification method for PME in soils [36]. Our method also reports a more comprehensive set of validation parameters and provides reliable analytical performance. Further optimization, such as the use of alternative acid modifiers or ion-pairing agents, may help achieve higher sensitivity and robustness in PMB quantification. While this UPLC-MS/MS method showed good applicability and reliable analytical performance, it was validated using only one loam soil. Differences in organic-matter content, mineral composition, or co-extracted interferents in other types of soils may still influence PMB recovery and matrix effects. These limitations do not undermine the usefulness of the method, but they do suggest caution when extrapolating its accuracy to diverse field soils. Broader soil-type validation would help further strengthen its applicability in heterogeneous agricultural environments.

The changes in PMB levels in soil after *P. polymyxa* inoculation were attributed to the combined effects of *P. polymyxa* biosynthesis and environmental degradation. In this study, *P. polymyxa* at the late exponential growth phase was used for soil inoculation to maximize polymyxin synthetase activity [54], which led to a rapid increase in PMB concentration within 7 days in the high-dose treatment group. In contrast, only minor increases in PMB levels were observed in the low- and medium-dose *P. polymyxa* groups, with concentrations remaining close to background values. With the colonization and proliferation of *P. polymyxa* in soil, the rate of PMB synthesis exceeded its environmental degradation rate, resulting in a continuous increase in PMB concentration, which peaked around day 14. Subsequently, from day 14 to 28, a decline in PMB concentrations was observed across all treatment groups, probably due to nutrient starvation that drove a portion of the *P. polymyxa* population into dormancy or sporulation [55]; causing PMB synthesis to fall below its degradation in the latter phase. Studies have shown that the half-life of PME in sandy loam increases with its initial concentration [36]. The differences in the magnitude of PMB decline among treatment groups from day 14 to 28 in this study are consistent with this reported pattern, which suggests that PMB exhibits concentration-dependent environmental persistence. Previous studies have reported that degradation half-lives (DT_50_) of antibiotics in agricultural soils vary widely. β-lactams such as amoxicillin degrade rapidly, with DT_50_ values below 1 day [56], whereas tetracyclines and macrolides (e.g., chlortetracycline, azithromycin) can persist for several tens to hundreds of days [57,58]. And some fluoroquinolones last even longer under certain conditions [59]. Based on the first-order degradation curves reported by Peng et al. [36], and assuming no further synthesis in soil, 0.3 mg/kg of PMB in sandy loam would be expected to dissipate within roughly 11 days, indicating a moderate level of persistence. According to the pharmacokinetic principles, a compound is generally considered eliminated after about five DT_50_ [60]. In this study, PMB persisted in soil well beyond the timeframe expected from conventional first-order degradation kinetics, which reflects ongoing synthesis by *P. polymyxa* rather than a reduced degradation rate. Although PMB concentrations gradually declined over the 28-day period, repeated application of *P. polymyxa* in agricultural practice and the long-term persistence of its spores in soils could lead to sustained PMB release into soil. This may impose prolonged polymyxin selective pressure on soil microbiota, with ecological risks potentially extending far beyond the 28-day temporal scope of our experimental observation. It should also be noted that the soil inoculation experiment used in this study represents a simplified system, and PMB behavior may differ in field soils with more complex microbial communities. Field conditions also vary in soil structure and moisture dynamics, which can influence PMB persistence. These factors may limit the direct extrapolation of our temporal patterns to real agricultural soils.

Finally, we assessed the ecological risks of PMB in soil following *P. polymyxa* inoculation to evaluate its environmental safety and potential contribution to AMR. The RQ method has been effectively applied to assess the ecological risks of antibiotics or ARGs in diverse environmental matrices, such as soil and aquatic systems [44,61]. To our knowledge, this study represents the first attempt to evaluate the ecological risk of PMB in soil. Due to the lack of available PNEC_soil_ data for PMB, this value was estimated using the equilibrium partitioning method recommended by the TGD of the European Commission [44]. In this method, PNEC_soil_ was calculated as follows: PNEC_soil_ = PNEC_water_ × *K_soil-water_*, where *K_soil-water_* was derived from the experimentally determined adsorption–desorption behavior of PME in loam soil reported by Peng et al. [36]. We adopted this approach because PMB and PME share the same peptide backbone, and PME is currently the closest available analog with reported soil-water partitioning data. This provides a reasonable basis for estimating the PNEC_soil_ value required for subsequent RQ calculation. However, PMB and PME differ in hydrophobicity and soil adsorption strength, and the physicochemical properties of the loam soil used in Peng’s study are not identical to those in our experiment. Therefore, the estimated PNEC_soil_ may deviate slightly from the actual value. To reduce this discrepancy, future studies should measure *K_soil-water_* values accurately for specific soil types. The RQ data derived in this study offer valuable insights for ecological risk assessment of PMB in soil. Although the measured PMB concentrations in this study did not reach the conventional toxicity-based risk threshold (RQ ≥ 1), previous studies have confirmed resistance selection may occur at concentrations below the PNEC [62]. Therefore, we suggest that the RQ value should not be used as the sole indicator for ecological risk assessment of PMB, and the potential risk of polymyxin-associated AMR at sub-PNEC levels should not be overlooked.

Thus, using the UPLC-MS/MS method established in this study, *P. polymyxa* was identified to be a significant source of PMB in soil, with its biosynthesis contributing to the persistence of PMB following inoculation. Evidence suggests that polymyxins may alter microbial structure and function in a dose-dependent manner and contribute to the enrichment of ARGs (e.g., *sugE*, *czcA*) and mobile genetic elements (MGEs) (e.g., *int1*, *IS26*) in the soil [22,23,47,63,64]. These shifts may disrupt the homeostasis of soil microbes and enhance the environmental persistence and mobility of resistance genes, thereby increasing the likelihood of AMR dissemination. Future research should validate these findings under field conditions and explore the long-term impact of *P. polymyxa* on soil microbiota and resistance gene dynamics.

## 5. Conclusions

In the present study, we developed a sensitive UPLC-MS/MS method to quantify PMB concentrations in soil. This method was applied to monitor the temporal dynamics of PMB in soil after *P. polymyxa* inoculation. As an efficient analytical tool, it enables accurate quantification of PMB from various sources in complex soil matrices, particularly in cultivated farmlands. The method also provides methodological support for future studies on the behavior and transformation of PMB in soil systems. We also established a preliminary framework for assessing the ecological risks of PMB in soil using the RQ method. The findings underscore the need for evaluation of the long-term environmental impacts associated with *P. polymyxa* application, especially the emergence, enrichment, and persistence of PMB-resistant bacteria in soil environments. These insights support more informed and sustainable use of *P. polymyxa* in agricultural soils.

## Figures and Tables

**Table 1:** Calibration curve, determination coefficient (R^2^), limit of detection (LOD), and limit of quantification (LOQ) of the UPLC-MS/MS method for PMB in soil matrix.

Matrix	Linear Equation	R^2^	LOD (ng/g)	LOQ (ng/g)
Soil	y = 37777x − 647896	0.9990	0.86	2.12

**Table 2:** Accuracy, precision, recovery, and matrix effect of the UPLC-MS/MS method for PMB in soil matrix.

Matrix	Spiked Level (ng/g)	Intra-Day (*n* = 3, %)		Inter-Day (*n* = 9, %)		Recovery (%)	Matrix Effect (%)
		Accuracy	RSD	Accuracy	RSD		
Soil	10	96.54	1.88	99.03	3.33	86.12 ± 1.61	90.2 ± 2.59
	50	98.47	3.15	97.09	2.63	83.58 ± 3.07	89.9 ± 1.42
	200	104.47	2.77	100.28	3.84	88.83 ± 1.48	87.7 ± 2.38

## Data Availability

The original contributions presented in this study are included in the article/Supplementary Material. Further inquiries can be directed to the corresponding author(s).

## Supplementary Materials

The following supporting information can be downloaded at: https://www.mdpi.com/article/10.3390/biom15121694/s1, Figure S1: MS/MS spectra of four polymyxin subtypes obtained in MRM mode (collision energy = 30 eV): (a) Polymyxin B1 (*m*/*z* 602.25 > 100.80), (b) Polymyxin B2 (*m*/*z* 595.40 > 101.25), (c) Polymyxin E1 (*m*/*z* 585.85 > 101.20), and (d) Polymyxin E2 (*m*/*z* 578.95 > 101.00); Figure S2: Calibration curve of PMB in soil matrix obtained by the UPLC-MS/MS method.

## Abbreviations

The following abbreviations are used in this manuscript:

AMR	Antimicrobial resistance
ARB	Antibiotic-resistant bacteria
ARG	Antibiotic resistance gene
Half-life	DT_50_
HGT	Horizontal gene transfer
HPLC-UV	High-performance liquid chromatography-ultraviolet
LB	Luria–Bertani
LC-MS	Liquid chromatography-mass spectrometry
LC-MS/MS	Liquid chromatography-tandem mass spectrometry
LOD	Limit of detection
LOQ	Limit of quantification
MDR	Multidrug-resistant
ME	Matrix effects
MRM	Multiple reaction monitoring
PBS	Phosphate-buffered saline
PMB	Polymyxin B
PME	Polymyxin E
PNEC	Predicted no-effect concentration
*P. polymyxa*	*Paenibacillus polymyxa*
RQ	Risk quotient
SPE	Solid-phase extraction
UPLC-MS/MS	Ultraperformance liquid chromatography-tandem mass spectrometry
XDR	Extensively drug-resistant
