# Supplementary material for: Development of a UPLC-MS/MS Method for Tracking Polymyxin B Dynamics in Soil Inoculated with Paenibacillus polymyxa
Source: Biomolecules. 2025 Dec 4;15(12):1694. doi: 10.3390/biom15121694 (PMC12730311; doi:10.3390/biom15121694)
Supplement: Supplementary file 1 [file biomolecules-15-01694-s001.zip › biomolecules-3987745-supplementary.pdf]

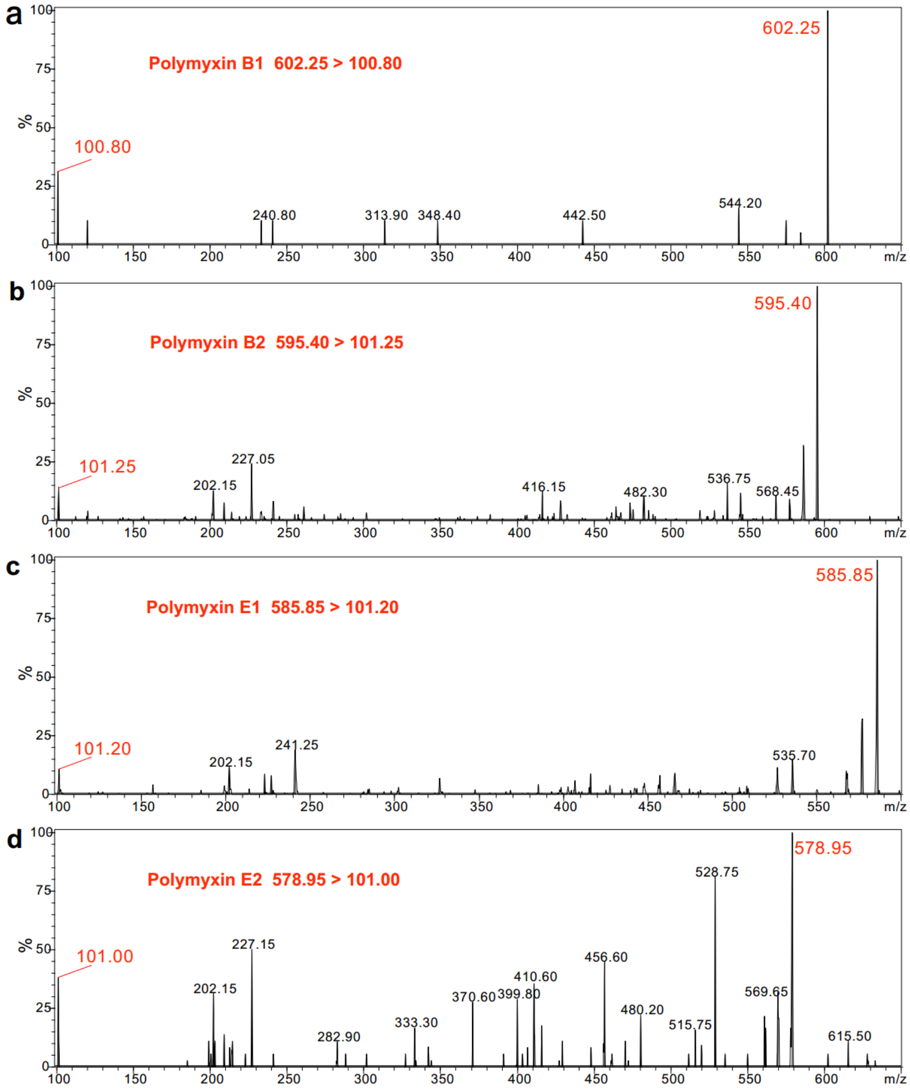

Figure S1. MS/MS spectra of four polymyxin subtypes obtained in MRM mode (collision energy = 30 eV): (a) Polymyxin B1 (m/z 602.25 > 100.80), (b) Polymyxin B2 (m/z 595.40 > 101.25), (c) Polymyxin E1 (m/z 585.85 > 101.20), and (d) Polymyxin E2 (m/z 578.95 > 101.00).

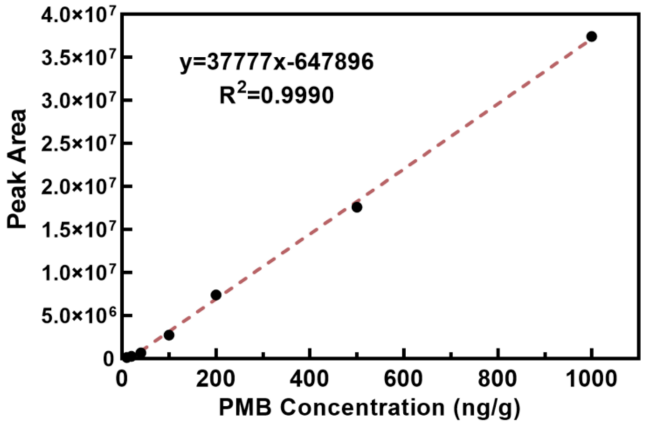

Figure S2. Calibration curve of PMB in soil matrix obtained by the UPLC-MS/MS method.
